# Deaths related to the use of diarylethylamines, with a focus on the United Kingdom: A systematic review and case series report

**DOI:** 10.1177/02698811251349203

**Published:** 2025-07-01

**Authors:** John Martin Corkery, Caroline Copeland, Fabrizio Schifano

**Affiliations:** 1 Hertfordshire Medical School, University of Hertfordshire, Hatfield, UK; 2 King’s College London, Institute of Pharmaceutical Sciences, London, UK; 3 King’s College London, National Programme on Substance Use Mortality, London, UK

**Keywords:** Diarylethylamines, diphenidine, ephenidine, methoxyphenidine, deaths, United Kingdom (UK)

## Abstract

**Background:**

Diarylethylamine drugs possess dissociative properties. These emerged as drugs of misuse, with reports of strong addictive potential, high tolerance and compulsive intake.

**Aims:**

Since one of these drugs, diphenidine, was added to the 1971 Convention on Psychotropic Substances, the United Kingdom (UK) had to consider its control. The Advisory Council on the Misuse of Drugs 2023 advice included toxicity and mortality involving this and related molecules. Relevant mortality data were collated to understand the international and UK situations.

**Methods:**

A systematic review was employed: PubMed, Scopus and Google Scholar searches were conducted on 29/30 August 2022 using the terms ‘overdose’, ‘death’, ‘fatal*’, ‘toxic*’, ‘poison*’ with molecules’ chemical names. UK Mortality Registers (MRs) provided statistical data. The Scottish MR and National Programme on Substance Use Mortality provided case-level information.

**Results:**

Eleven studies were identified. Most decedents were male. The mean death age was 35.3 (range: 17–55) years. Death was commonly from polysubstance poisoning. Globally, 48 deaths involved these drugs (Europe *n* = 40). Of these, 37 occurred in the UK in 2014–2019. Key characteristics were as follows: male (91%); White (95%), mean age 37.2 (range: 19–65) years; drug use history (72%). Most deaths (89%) were accidental from acute drug toxicity (92%). Diphenidine/methoxyphenidine (MXP) was implicated with other substances (opioids/opiates, benzodiazepines, stimulants) in 66% of cases.

**Conclusions:**

Most deaths were accidental – thus preventable. One-third of deaths involved MXP/diphenidine alone – suggesting they are relatively toxic. Diarylethylamines deaths are rare. These molecules remain available – deaths could occur.

## Introduction

### Context

In January 2022, the United Kingdom’s Advisory Council on the Misuse of Drugs (ACMD) was commissioned by the then Home Secretary to advise upon the appropriate classification of diphenidine under the Misuse of Drugs Act 1971 and its scheduling under the Misuse of Drug Regulations 2001. The Home Office was obliged to control diphenidine following its addition to Schedule II of the Convention on Psychotropic Substances of 1971 during the 64th Commission on Narcotic Drugs meeting in April 2021 (ACMD, 2023a).

The ACMD took the opportunity to look at and provide advice on several other related dissociative diarylethylamine substances – ephenidine, methoxphenidine, fluorolintane and isophenidine. These five molecules are known by several names: diphenidine – DPD, 1-(1,2-diphenylethyl)piperidine, 1,2-DEP and DND; ephenidine – NEDPA and EPE; fluorolintane – 2-FPPP and 2-F-DPPy; isophenidine – NPDPA and isopropylphenidine; methoxphenidine – MXP, 2-MXP, methoxyphenidine, methoxydiphenidine, 1-[1-(2-methoxyphenyl)-2-phenylethyl]piperidine, piperidine, 1-(1-(2-methoxyphenyl)-2-phenylethyl)-, (±)- 1-(2-methoxyphenyl)-2-phenyl-1-(piperidine-1-yl)ethane2-MeO-Diphenidine.

As part of their consideration, the Council examined physical harms including toxicity and deaths. The first two authors were members of the ACMD’s Working Group and led on assembling information on deaths related to the use of these substances, especially any occurring in the United Kingdom (UK). They wrote a summary report, which was included in the ACMD’s published report (ACMD, 2023b). This article presents the more detailed research underlying that summary.

### Diarylethylamines and their uses

Compounds from the 1,2-diarylethylamine class have been investigated for clinical use in the treatment of depression, epilepsy, pain and neurodegenerative disease (Wallach et al., 2016).

However, at the time of writing, in Europe and North America, there are no approved industrial, medicinal or veterinary uses for these specific molecules.

Diarylethylamines have dissociative properties akin to those of ketamine and phencyclidine/phenylcyclohexyl piperidine (PCP); this is particularly true for derivatives of lefetamine (N,N-dimethyl-1,2-diphenylethanamine; ACMD, 2023b). They are one of the two structurally distinct chemical classes that have emerged in the last decade or so as Novel Psychoactive Substances (NPS) – the other being β-keto-arylcyclohexylamine analogues of ketamine, such as methoxetamine, deschloroketamine, etc. For example, diphenidine – although first synthesised in 1924 – emerged as a ‘research chemical’ in 2013 (Wallach et al., 2015), along with MXP – first patented in 1989 (Morris and Wallach, 2014).

Typically, ‘dissociatives’ are labelled as psychedelics, whereas they are actually a sub-class of the hallucinogen class. Dissociative compounds are those which are described as detaching the user from reality and altering the perception of sight and sounds, but whilst ‘mind altering’ are thus not considered hallucinogens (Schifano et al., 2019).

The principal mode of action of diarylethylamines is acting as N-methyl-D-aspartate (NMDA) receptor antagonists, thereby generating dissociative effects (Kang et al., 2017). Reuptake of dopamine and norepinephrine is inhibited by diphenidine, ephenidine and MXP (Kang et al., 2017; Wallach et al., 2016). Like ketamine, these molecules affect dopamine transporter availability and hence dopamine transmission (Wallach et al., 2016).

Dissociative effects have been reported on social media platforms for these molecules (ACMD, 2023b; Morris and Wallach, 2014). The usual routes of administration appear to be inhalation and smoking; modes for the latter include mixing with herbal smoking products, vapourising over heated aluminium foil and electronic smoking devices (vapes). Desired effects occur more rapidly via inhalation/smoking (15–30 min) rather than insufflation. The length of effects (4–24 h) is determined by the amount utilised and length of use. Dosages for diphenidine, ephenidine and MXP are given by the ACMD (2023b: 6). The potential use of MXP for therapeutic purposes has also been discussed online (Van Hout and Hearne, 2015).

Online users fora warn about the strong addictive potential, high tolerance and compulsive intake of diphenidine, ephenidine and MXP (Wallach et al., 2016). Researchers also suggest that these substances probably have addictive potential (Eiden et al., 2018; Sahai et al., 2018). Indeed, Eiden et al. (2018) mention cases of withdrawal syndrome for diphenidine and MXP.

### Availability and prevalence

As with other NPS, the molecules considered here are easily available to purchase on the Internet, being advertised as ‘research chemicals’ and ‘not for human consumption’. Initially, they were marketed as legal replacements for other controlled molecules, especially acrylcyclohexylamines such as methoxetamine.

There are no prevalence estimates of the use of these five molecules. When the ACMD compiled its report, no cases of acute toxicity had been published related to or detections made of ephenidine, fluorolintane or isophenidine, whereas there were reports of acute toxicity and deaths involving the use of diphenidine and MXP (ACMD, 2023a). Analysis of anonymised ‘sample results’ from Welsh Emerging Drugs & Identification of Novel Substances (WEDINOS; https://www.wedinos.org/) by the ACMD (2023b: 15–18) demonstrated that there have been no samples processed by the WEDINOS where isophenidine or fluorolintane were detected. The other three molecules have shown up relatively rarely since 2013/4: diphenidine 8 in 2013/14–2015/16; ephenidine 9 in 2015/16–2016/17; MXP 26 in 2013/14–2016/17 and 4 in 2019/20 (ACMD, 2023b: 15). By November 2022, the UK Forensic Early Warning System had detected MXP in only two samples sent to them for analysis (both in 2020/21); none of the other molecules in this group were detected (ACMD, 2023b: 16).

### Pharmacological and toxicological effects

When analysed, some 1,2-diarylethylamines are potent antagonists of the NMDA receptor and inhibitors of monoamine transporters (Wallach et al., 2019). As NMDA receptor function is critical to central nervous system processes, their pharmacomodulation can precipitate dissociative effects (Wallach et al., 2019).

Clinical features of 1,2-diarylethylamines administration resemble those observed following stimulant consumption (e.g., hypertension, tachycardia) and also induce neuropsychiatric aberrations (e.g., hallucinations, sedation/drowsiness, confusion, paranoia and anxiety/agitation) – clinical features similar to those seen following the use of dissociative drugs such as ketamine (ACMD, 2023a).

There is little by way of toxicological information on these molecules, other than from case reports on the acute toxicity of diphenidine and MXP; these are summarised in the ACMD (2023b: 11–13) report. The reader is directed to that publication for further details, including each compound’s role as an NMDA antagonist.

### Study rationale

This group of dissociative substances had never been assessed in terms of whether they should be controlled under the UK’s primary drug legislation. The material presented here was assembled to inform this process.

### Aim and objectives

The overarching aim was to provide as much objective information as possible about deaths involving these specific substances. The first objective was to identify and summarise the existing information in the public domain about such events. The second objective was to identify and summarise what was known at that point about such instances, specifically in the UK.

## Methods and materials

Two principal methods were deployed to achieve these objectives. A systematic review of the extant academic literature, supplemented by searches of secondary data sources, was employed to identify and assess existing information on deaths relating to the use of these substances. Searches of existing specialist data sources accessible to the first two authors were utilised to identify such deaths that had occurred in the UK. Further details of the systematic searches can be found in the Supplemental materials.

### Data sources

The lead author and ACMD Secretariat contacted the three UK General Mortality Registers (GMRs) for information on deaths involving this group of substances: National Records of Scotland (NRS); Northern Ireland Statistics and Research Agency and the Office for National Statistics (ONS), which covers England and Wales. The GMRs hold data derived from Medical Certificates of Cause of Death.

Anonymised data provided by NRS to the lead author were examined. The second author has access to the data that have been voluntarily provided by coroners in England, Wales and Northern Ireland since 1997.

The Cause of Death and Toxicology fields of the two datasets were searched using the numerical code assigned to the specific substances, for example, ‘diphenidine’, ‘ephenidine’, ‘methoxphenidine’ and ‘MXP’ and these names, respectively.

### Ethical considerations

The ethics committee of the second author’s institution re-confirmed in May 2024 that the coroner’s database does not require ethics review, as all subjects are deceased. Only aggregated data are presented below.

## Results

The results of the systematic review of literature and searches of specialist databases are briefly summarised in the ACMD report (ACMD, 2023b: 8). Here, they are presented separately.

### Systematic review of academic literature

**Figure 1.**
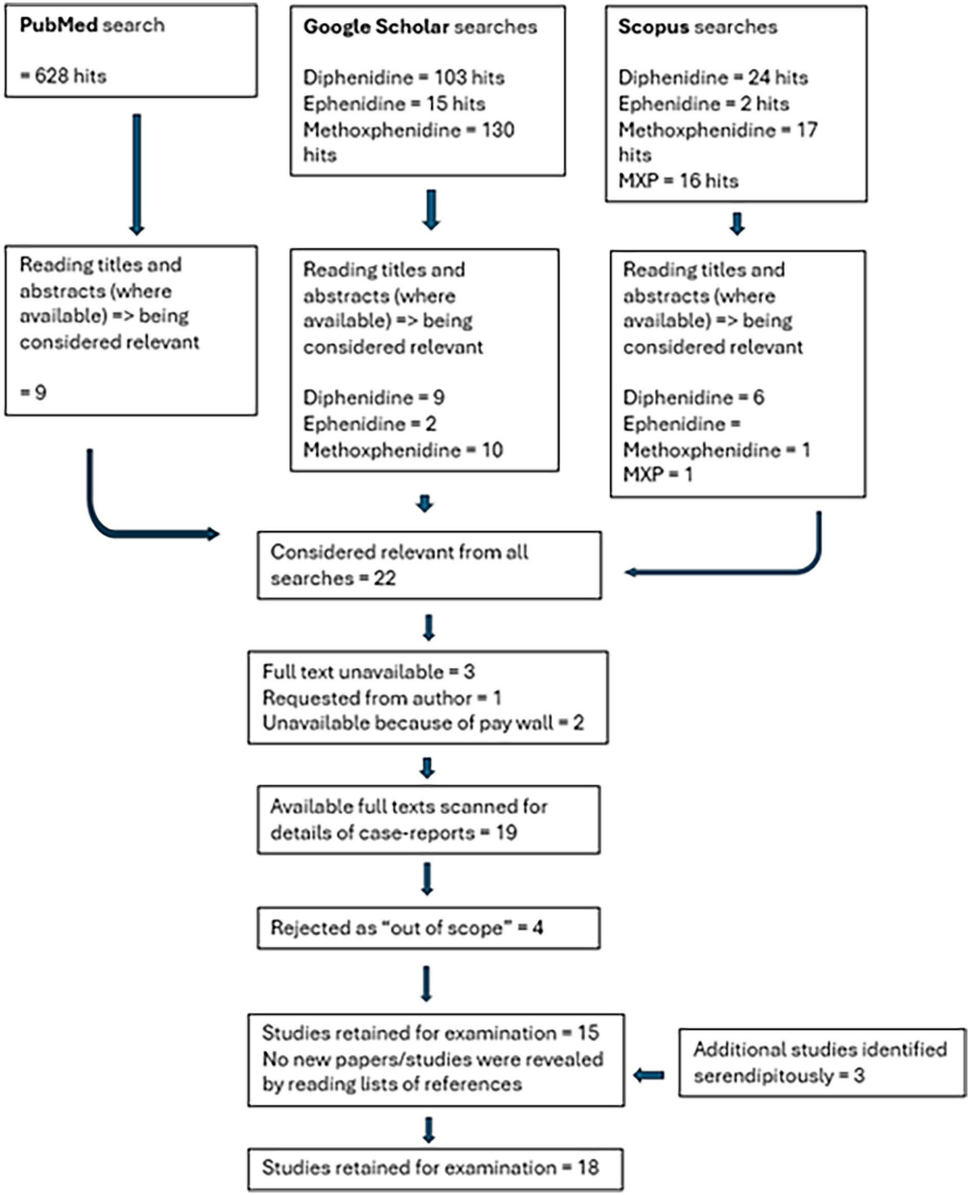
Flowchart of systematic literature review.

The overall process of the literature searches and the selection processes is outlined in Figure 1.

Of the 18 papers/studies (that were available) regarded as relevant, only 7 contained references to papers containing case details or case reports. The remaining 11 papers/studies provide information on 4 case details and 9 case reports.

Other sources surveyed were as follows: EMCDDA (European Monitoring Centre for Drugs and Drug Addiction) risk assessments (none); EU-MADNESS (EUropean-wide, Monitoring, Analysis and knowledge Dissemination on Novel/Emerging pSychoactiveS) cases (1 Hungarian case and 6 NPSUM cases); Scottish Fatal Accident Inquiries (none) and the Chief Coroners website Regulation 28 Report (1 case).

Summary details of the published sources are given in Table 1. Five are from Japan, two from England and Wales, and one each from France, Germany and the USA. Five relate only to diphenidine, four to MXP and one covers both molecules. No source mentions ephenidine. The year of death was only ascertainable, using personal contacts, for the English and Welsh sources. The papers were published between 2015 and 2018.

**Table 1.** Summary details of selected papers/studies.

Authors and year of publication	Year(s) of death	Country	Type of report	Index substance(s) mentioned
Elliott et al. (2015)	(First half of 2014 – personal communication)	England and Wales	3 case reports	Methoxphenidine (3)
Elliott et al. (2018)	(First half of 2014 – personal communication)	England and Wales	3 case reports (same as previous row)	Methoxphenidine (3)
Hasegawa et al. (2015)	Not stated	Japan	1 case report	Diphenidine
Minakata et al. (2016)	Not stated	Japan	1 case report (same as previous entry)	Diphenidine
Kudo et al. (2015)	Not stated	Japan	1 case report	Diphenidine
Grumann et al. (2017)	Not stated	Germany	1 case report	Methoxphenidine
Logan et al. (2017)	Not stated	United States	4 case details	Diphenidine (1), methoxphenidine (MXP) (3)
Kinoshita et al. (2017)	Not stated	Japan	1 case report	Diphenidine
Saint-Martin et al. (2017)	Not stated	France	1 case report	Methoxphenidine (MXP)
Kusano et al. (2018)	Not stated	Japan	1 case report	Diphenidine

Case details from combined sources are given in Table 2. In summary, the number of deaths by country was in descending order: England 5; Japan 4; USA 4; Wales 2; France 1; Germany 1; Hungary 1. Most decedents were male (15/18). Age at death ranged from 17 to 55 (mean 35.3, mode 34) years.

**Table 2.** Details of case reports from selected papers/studies, additional cases found via Google searches and the EU-MADNESS project.

Source	Type of source	Country	Gender	Age (years)	Circumstances of death	Autopsy findings and cause of death	Manner of death	Toxicology levels	
								Dissociative substance(s)	Other substance(s)
Elliott et al. (2015, 2018)	Peer-reviewed journal article	Wales	M	34	Found dead at home. Evidence of drug paraphernalia, including white powder in clear bags that was later identified to be ‘methoxphenidine’ (isomer not distinguished) by a police drug laboratory. The drug had been purchased online from a ‘research chemical’ company	At autopsy, he was found to have an enlarged heart and hypertensive heart disease with no other contributory findings. Based on the pre-existing heart disease and potential cardiac effects of 2-MXP along with no other significant pathological findings, 2-MXP was considered to have likely contributed to death, even in the presence of other drugs and was cited in the cause of death: “methoxyphenidine [sic] use and hypertensive heart disease”. (Coroners’ database case)	Not known	2-MXP at 24.0 mg/L in post-mortem femoral blood; also detected in urine	Prescription drugs (mirtazapine, lamotrigine and citalopram) at therapeutic concentrations
Elliott et al. (2015, 2018)	Peer-reviewed journal article	England	M	34	Found dead at home. Had a medical history of epilepsy, attention deficit hyperactivity disorder and social anxiety. Had been prescribed levetiracetam, dexamphetamine and diazepam. A sachet labelled ‘Methoxphenidine 2 g’ was found in his pocket	At autopsy, he was found to have a moderately enlarged heart and mild atheroma with no other contributory findings. The low concentrations of other drugs do not suggest excessive use of these; and whilst non-compliance with anti-epileptic medication was considered circumstantial and other evidence excluded this as a factor. With no other significant pathological findings, 2-MXP was considered to have likely contributed to death, even in the presence of other drugs and was cited (albeit on the balance of probability) in the cause of death: “probable methoxphenidine toxicity” (Coroners’ database case).	Not known	2-MXP (Methoxphenidine) at 2.0 mg/L in post-mortem femoral blood; also detected in urine	Prescription drugs (diazepam and quinine) at therapeutic concentrations. Neither levetiracetam nor dexamphetamine were detected
Elliott et al. (2015, 2018)	Peer-reviewed journal article	England	M	38	Found dead on a road, having jumped or fallen from a road bridge, sustaining fatal injuries. Had a medical history of schizophrenia. No other information was available	The cause of death was due to multiple injuries following the fall, and the inquest conclusion was ‘suicide whilst suffering from a mental illness’. The circumstances and stated cause of death conclude that 2-MXP was not the direct cause of death. Whilst an altered state of mind as a result of 2-MXP use could be a consideration, the pre-existing mental health history was considered a primary factor (Not reported to coroners’ database).	Suicide	2-MXP at 1.36 mg/L in post-mortem femoral blood; also detected in urine	Prescription antipsychotic drug risperidone at a therapeutic concentration
Hasegawa et al. (2015), Minakata et al. (2016)	Peer-reviewed journal articles	Japan	M	30	The decedent’s body was found in his car, located in a corner of a supermarket car park. He was lying in the prone position with his head on the front passenger seat and with his left leg on the driver’s seat. He wore trousers, but was naked from the waist up. He held a disposable lighter in his right hand. Just below his chest, an open package of herbal blend labelled as “Herbal Incense. The Super Lemon” was found. A battered, empty coffee can was found on the driver’s seat; on the surface of the flattened can, fire debris and herb ash were observed, suggesting that he had inhaled the smoke of the herbal blend after using the lighter to burn the material on the flattened part of the empty coffee can. Another small package of herbal blend without any label or brand name, which was also open, was found in the middle part of the back seat of the car. Upon medical examination at the scene, no criminality was suspected for the deceased; the body was relatively fresh, and no injuries were found on the body surface. An autopsy took place about 3.5 days after death. Macroscopically, there was livor mortis associated with vibices for wide areas of the body surface. There was extensive post-mortem epidemic desquamation. There were no serious injuries related to the death of the victim. Internally, there were a few subcutaneous haemorrhages in the left lower leg	Analysis of the herbal specimens found at the scene strongly suggested two synthetic cannabinoids, AB-CHMINACA and 5-fluoroAMB. The extract solution obtained from the package labelled “Herbal Incense. The Super Lemon” revealed diphenidine. Therefore, attention was focussed on the identification and quantitation of AB-CHMINACA, 5-fluoro-AMB and diphenidine in body fluids and solid tissues obtained from the victim. Due to the high concentrations of diphenidine, particularly in the adipose tissues, it was concluded that this substance was the one responsible for causing death	Not known	Diphenidine (ng/ml or g): femoral blood: 715 ± 28.3; right heart blood: 707 ± 62.9; left heart blood: 923 ± 62.8; urine: 376 ± 23.7; brain: 1550 ± 49.1; heart muscle: 2070 ± 73.5; lung: 1600 ± 13.9; liver: 2960 ± 34.0; spleen: 1300 ± 31.9; kidney: 2510 ± 32.9; pancreas: 1910 ± 38.1; abdominal subcutaneous adipose tissue: 11,100 ± 1120	5-FluoroAMB (g): abdominal subcutaneous adipose tissue: 18.7 ± 1.10. Not detected in urine and blood samples. AB-CHMINACA (ng/ml or g): brain: 15.6 ± 0.39; heart muscle: 20.0 ± 3.00; lung: 8.02 ± 0.71; liver: 21.2 ± 1.30; spleen: 7.55 ± 0.18; kidney: 24.7 ± 1.63; pancreas: 38.9 ± 4.55; abdominal subcutaneous adipose tissue: 24.8 ± 2.48. Not detected in urine and blood samples
Kudo et al. (2015)	Peer-reviewed journal article	Japan	F	30s	Found dead in the supine position on a bed. She had talked to her former boyfriend by phone 3 days prior, complaining that she had a headache. Subsequently, he was unable to contact her. Considerable amounts of ‘aroma liquid’ and ‘bath salt’ products and hypnotic drug tablets were found scattered on the floor near the bed. 5 aroma liquid bottles named “High H”, “High X” and “TATOO” and the residues in 11 plastic tubes with packages named “Mona Lisa 4”, “Christ 2”, “Girls ROOM POWDER” and “Mozart”: contained PV9, 4-methoxy PV8, 4-methoxy PV9 and diphenidine	Autopsy performed approximately 4 days after estimated time of death. Height 164 cm, weight 50 kg. There were no apparent external injuries that could be related to the death of the decedent. Internal examination showed only pulmonary congestion and oedema. The cause of death was determined to be poisoning by 3 types of cathinone drugs, 4-methoxy PV8, PV9, and 4-methoxy PV9, and diphenidine under the influence of 3 benzodiazepines and alcohol	Not known	Diphenidine (µg/ml): femoral bl 1.38; right heart bl 1.48; left heart bl 1.68; urine 0.843; gastric 94.7	4-methoxy PV8, PV9, 4-methoxy PV9, triazolam, flunitrazepam, nitrazepam, a-hydroxytriazolam, 7-aminoflunitrazepam and 7-aminonitrazepam measured. Three benzodiazepines (nitrazepam, flunitrazepam and triazolam) in femoral blood were within the therapeutic range; a relatively high concentration of 7-aminonitrazepam was detected. Blood alcohol concentration = 1.52 mg/ml
Grumann et al. (2017)	Peer-reviewed journal article	Germany	M	21	Found dead in his bathtub with his upper body completely covered by water. Decedent was a known drug user in the past and had been treated with psychotropic drugs due to deliberate self-injury	Typical signs of drowning, as well as superficial cuts crossing the inner sides of both wrists, were found during autopsy. A puncture site at the crook of the left arm was also found	Considering the post-mortem results, indicating a mixed intoxication including methoxphenidine and ethanol, an accidental death due to loss of consciousness with subsequent drowning seems plausible. However, suicide could not be excluded	Methoxphenidine was found at a concentration of 190 ng/ml in femoral blood; urine +	Femoral bl: lorazepam 5.7 ng/ml; delorazepam 54 ng/ml; amphetamine 64 ng/ml; 4-fluoroamphetamine (4-FA) 2.1 ng/ml. Urine uptake of amphetamine, 4-FA, diclazepam (indicated by additional finding of lormetazepam). The blood alcohol concentration was 0.93‰
Logan et al. (2017)	Peer-reviewed journal article	USA	M	30	Found dead in a car with a lighter in hand and an open package of “Herbal Incense the Super Lemon”	No significant findings or obvious cause of death. AB-CHMINACA and diphenidine implicated in death	Not known	Diphenidine blood 707–923 ng/ml; urine: 376 ng/ml; tissue: 1300–11,100 ng/g	AB-CHMINACA tissue: 7.6–38.9 ng/g + Adipose tissue: 18.7 ng/g 5F-AMB
Logan et al. (2017)	Peer-reviewed journal article	USA	M	34	Found dead at home with powder nearby, identified as MXP	Enlarged heart, hypertensive heart disease	Not known	Methoxphenidine femoral blood: 24,000 ng/g	
Logan et al. (2017)	Peer-reviewed journal article	USA	M	34	Found dead at home with a history of epilepsy, powder found in the pocket	Moderately enlarged heart, mild atheroma	Not known	Methoxphenidine femoral blood 2000 ng/g	
Logan et al. (2017)	Peer-reviewed journal article	USA	M	38	Died after jumping from a bridge, had schizophrenia	(Fall from height)	Not known	Methoxphenidine femoral blood: 1360 ng/g (alternate cause of death)	
Kinoshita et al. (2017)	Peer-reviewed journal article	Japan	F	20s	Found dead, lying face down on her bed. Numerous empty packets of prescription drugs and a package of new psychoactive substances with an implement for smoking were nearby. Subsequent police investigations revealed that the deceased had been prescribed medication for insomnia	Autopsy revealed slight abrasions on the dorsal surface of both hands, but they were not considered contributory to the cause of death. No findings of natural disease were observed. Height 157 cm, weight 45 kg. Lungs were congested. Histological examination revealed marked congestion and oedema in the lungs. Based on the autopsy findings, the results of toxicological examination and the investigation by the authorities, it was concluded that multiple drug ingestion, including benzodiazepines, histamine H1 receptor antagonist and new psychoactive substances, led to death due to their combined toxicity	Not known	Diphenidine blood (unspecified) 0.073 µg/ml. Diphenidine was at about 1-10th of the fatal range (>0.71 µg/ml)	Blood (unspecified) 7-aminoflunitrazepam (metabolite of flunitrazepam) 0.086 µg/ml, 7-aminonimetazepam (metabolite of nimetazepam) 0.027 µg/ml, chlorpheniramine 0.066 µg/ml. The toxic level of 7-aminoflunitrazepam; chlorpheniramine was slightly over the normal therapeutic range (0.003–0.017 µg/ml). Flunitrazepam, nimetazepam and nitrazepam were below the detection level
Saint-Martin et al. (2017)	Peer-reviewed journal article	France	M	55	Found dead in his house. Drug paraphernalia was collected near the body: a parcel that was sent from abroad was found on a table, containing 5 labelled single-dose packets with white powder indicating either synthetic cannabinoids (MMB-CHMINACA) or other designer drugs (methoxphenidine – MXP, methiopropamine – MPA, 3-fluorophenmetrazine – 3-FMP), as well as several lighters, a broken pipe with brown residue and a glass with white powder	The autopsy was unremarkable, except for an intense and diffuse congestion and pulmonary oedema. Macroscopic and microscopic observations failed to reveal either external injuries or endogenous diseases. The packets, the pipe and the glass contents were found to contain multiple psychoactive substances: ADB-CHMINACA (one packet), MXP (one packet), MPA (one packet), 5F-APINACA, AB-FUBINACA, FUBIMINA, MMBCHMINICA, MPA and 3-FPM (one packet), MMB-CHMINACA and FUBIMINA (the pipe), ethylphenidate, 5F-APINACA, AB-FUBINACA, AB-CHMINACA, FUBIMINA, THJ-018, MMB-CHMINACA, 3-FPM, MPA and MXP (the glass). Death was attributed to combined drug intoxication	Not known	MXP: peripheral blood 0.081 μg/ml; urine 0.285 μg/ml	Peripheral blood: MMB-CHMINACA; ADB-CHMINACA; oxycodone: 0.104 μg/ml; oxazepam 0.11 μg/ml; MPA 2 ng/ml; 3-FPM: 11.4 μg/ml; alimemazine 0.09 μg/ml. The same drugs, together with zopiclone, were also detected in the urine; 3-FMP 32 μg/ml
Kusano et al. (2018)	Peer-reviewed journal article	Japan	M	53	Decedent had been absent from his office; he was found dead in his apartment. An open package of a branded herbal blend (“Heart Shot BLACK”) known to contain synthetic drugs of abuse, along with several pipes suspected to have been used for smoking the herbal blend, were found near him. Analysis of the package’s contents by a local police forensic science laboratory found it contained 5F–ADB. No other drugs of abuse were detected in the herbal product	Autopsy conducted approximately 2 days after death. There were no apparent specific injuries or internal findings that could have caused the decedent’s death. While the investigators could not definitively conclude that 5F–ADB and diphenidine intoxication was the fatal cause, it is considered highly probable, since no other competing cause(s) of death were found	Not known	Diphenidine right heart blood = 12 ± 2.6 ng/ml	5F–ADB right heart blood = 0.19 ± 0.04 ng/ml
EU-MADNESS project (UH and NPSUM)	Notification from research collaborators (Semmelweis University, Department of Forensic and Insurance Medicine)	Hungary	M	17	Found on the street (2014)	Drug overdose	Not known	PM blood – 2-MeO-diphenidine/MXP 1006 ng/ml, diphenidine 2,9 ng/ml; PM urine – 2-MeO-diphenidine/MXP 505.7 ng/ml	PM blood – ADB-Chminaca 0.7 ng/ml
Regulation 28 report	Report from coroner to ministry of justice (Courts and Tribunals Judiciary, 2015)	England	F	37	The decedent, who had a history of drug abuse, was found unresponsive and not breathing at their home address by a parent (2015). Paramedics and pronounced life extinct. In addition to prescribed medication, the police found a quantity of white powder labelled “Methoxyphenidine” [sic]. She was known to buy substances online	There were no post-mortem findings that could explain the decedent’s sudden death. The medical cause of death given as “Methoxyphenidine and Cocaine Toxicity”. (Coroners’ database case)	Drug-related death	Methoxphenidine	Cocaine
Google search	Media report (Goddard, 2015)	Wales	M	19	Medical history includes fibromyalgia, anxiety, previous overdoses and suicidal thoughts. Medication prescribed included temazepam, sertraline, pregabalin, co-codamol, lansoprazole and vitamin B12. The history of using ‘legal highs’ and had been referred to a local mental health team for treatment; a letter asking him to set up an appointment arrived 4 days after he died. In possession of a gram of heroin. Clonazolam was also found at the scene (2014)	Died after taking a combination of heroin, etizolam and diphenidine. Cause 1a “Opiate toxicity in combination with etizolam and diphenidine” (Coroners’ database case).	Drug-related death	(Diphenidine + metabolites bl; ur) – not quantified	Heroin (morphine 103 µg/l bl; ur; M3G 327 µg/l bl; ur; M6G 56 µg/l bl; ur; codeine bl; ur; noscapine bl; ur; papaverine bl; ur; 6-MAM bl; ur). Etizolam + metabolite bl; ur. Propranolol bl; ur. Diphenhydramine ur
Google search	Media report (Sussex World, 2014)	England	M	45	Found by friends in the bedroom of the mother’s home. Believed to have taken a drug overdose and suffered a cardiac arrest shortly after the arrival of paramedics. Medical history of depressive disorder and was prescribed propranolol. Decedent had access to diphenidine, etizolam and ‘acid’ (assumed to be referring to LSD). A couple of partially drunk bottles of champagne and a packet of powdered drug (diphenidine) were found at the address. Probably bought online (2014)	Cause of death: 1a Diphenidine toxicity, 2 Ethanol toxicity. (Coroners’ database case)	Accidental	Diphenidine – blood: 2.4 mg/dl	Blood – alcohol 165 mg/dl
Google search	Media report (Winter, 2016)	England	M	46	Decedent took formerly ‘legal highs’ in a bid to cope with his mental health problems (including OCD and a form of ADHD). Found slumped on a sofa at his home address by a neighbour. Two packets of white powder were found in his trousers pocket: <0.5 g of 3-fluorophenmitrazine and 2.43 g of methoxphenidine (2016)	Death as a consequence of abuse of drugs (Not on Coroners’ database)		Methoxphenidine	

Where known (*n* = 17), the majority (11) were found dead at home, with a further case being found dead at a parent’s house. Two fatalities occurred following falls/jumps from bridges; two in parked cars, and one decedent was found dead in a street. One of the fatalities found at home appears to have drowned in a bath. Based on evidence from the *locus* of death, smoking or inhalation was the likely route of administration.

Detailed autopsy/pathology findings were lacking in many cases. The key findings of note were as follows: enlarged heart and hypotensive heart disease (2); enlarged heart and mild atheroma (2); multiple injuries (2) and signs of drowning (1).

The commonest cause of death was poisoning/toxicity/intoxication – typically involving other substances, including alcohol. There were only two cases where one of these dissociative substances (MXP) was the sole substance implicated; one was contributed to by hypertensive heart disease. As already noted, two deaths were caused by falls/jumps from height, leading to multiple injuries. The manner of death was only available for five cases. Two were regarded as ‘drug-related’, one as accidental, one as suicide and one as accidental or possible suicide.

There were three cases where MXP was the sole drug mentioned/implicated in death and for which toxicology levels were available; these ranged from 1360 to 24,000 ng/g in femoral blood. Other levels given for the substance in combination with other substances ranged from 1.36 to 24.0 mg/L. Femoral blood levels for diphenidine in combination with other substances were only available for two cases (0.138 and 0.715 mg/L).

A range of other substances were also reported in the post-mortem toxicology, many of which are mentioned in the cause of death: heroin, cocaine, amphetamine, synthetic cannabinoids; synthetic cathinones; prescription drugs, especially benzodiazepines and alcohol. This variety of substances reflects the plethora of psychoactive substances available for recreational use over the past decade, and the continuing trend of polysubstance use.

Overall, it can be confidently estimated that there have been at least 48 deaths involving these index drugs worldwide. The majority (*n* = 40) of these deaths occurred in Europe, with four being reported in the United States and four in Japan. In terms of Europe, fatalities were reported in France (1), Germany (1) and Hungary (1). However, the majority (*n* = 37) of the cases were reported in the UK.

Based on the published case reports, information provided by ONS and NRS, and the information published and/or provided by the coroners’ database, and Google searches, the authors can state that the reported deaths involving this group of diarylethylamines in the UK all occurred between 2014 and 2019, the majority happening in 2014–2016, with one occurring in 2019.

### UK figures

Communication by the lead author and the ACMD Secretariat with the three official UK GMRs and the latter’s responses/data provide the following information. No cases reported in Northern Ireland (up to the end of Q1 2022); seven cases in Scotland (including one where both diphenidine and MXP were implicated, occurred between January 2014 and March 2019); and the following from ONS in respect of England and Wales, as of 5 August 2022, there were 21 mentions of diphenidine and MXP, but none of ephenidine. A paper based on the coroners’ data reports a total of 4 detections involving diphenidine and 24 involving MXP between May 2013 and May 2019 (Deen et al., 2021); updated data are included in the analyses below.

Pooling all reports known to the first and second authors, they were aware of at least 37 deaths in the UK up to their submission to the ACMD (Table 3). ONS reports a maximum of 21 deaths registered in England and Wales to date; however, combining information from the coroners’ database, Elliott et al. (2015) and Google searches, the authors have identified at least 31 in these 2 countries during the same period. This discrepancy is probably due to differences in the quality and detail of information (including pathology and toxicology reports) provided by coroners compared to what is received by ONS. There may even be additional cases which have not been reported to the coroners’ database, given that at least one case found using Google searches was unknown to them, and that reporting to the coroners’ database is on a voluntary basis. Information was not necessarily available for all variables across all cases.

**Table 3.** UK deaths involving diphenidine and/or MXP by year of occurrence.

Year	Country										
	England		Wales		Scotland			United Kingdom			
	Molecule		Molecule		Molecule			Molecule			All
	Diphenidine	MXP	Diphenidine	MXP	Diphenidine	MXP	Diphenidine + MXP	Diphenidine	MXP	Diphenidine + MXP	
2014	1	8	1	1		2	1	2	11	1	14
2015	1	11		1				1	12		13
2016	1	5				3		1	8		9
2019					1			1			1
Total	3	24	1	2	1	5	1	5	31	1	37

Sources: Elliott et al. (2015), Coroners’ database, NRS, Winter (2016).

No cases in Northern Ireland.

### Socio-demographics

Information on socio-demographics was available for 35 NPSUM and NRS cases. Breakdowns by country are given in Table 4. The key high-level findings for Great Britain (i.e., England, Wales and Scotland) are as follows: the majority of cases involved males: 32/35 (91.4%); mean age was 37.2 (range: 19–65) years; where known (*n* = 19), the majority were of White ethnicity (94.7%); 54.2% were unemployed and 37.5% were employed; 53.6% lived with others and 40.7% lived alone; 72% had a history of drug use, 28% did not; 71.4% (20) had been prescribed drugs – antidepressants (7), benzodiazepines/‘Z’ drugs (6), gabapentinoids (5), opioids (3), beta-blockers (3), anti-psychotics (2), anti-epileptics (1) and amphetamines (1).

**Table 4.** Socio-demographic characteristics of decedents where diphenidine and/or MXP was implicated, by country.

Characteristic	Country			
	England (*N* = 25)	Wales (*N* = 3)	Scotland (*N* = 7)	Great Britain* (*N* = 35)
Gender	Male 23; female 2	Male 3; female 0	Male 6; female 1	Male 32; female 3
Age (years)	Mean 36.3, range 22–56	Mean 34.7, range 19–51	Mean 41.4, range 20–65	Mean 37.2, range 19–65
Ethnicity	White 17; Black 1; not known 7	White 1; not known 2	Not available 7	White 18; Black 1; not known 9; not available 7
Employment status	Employed 9; student 1; retired 1; unemployed 12; not known 2	Unemployed 1; not known 2	Not available 7	Employed 9; student 1; retired 1; unemployed 13; not known 4; not available 7
Living arrangements	Alone 11; with others 12; student halls 1; not known 1	With others 3	Not available 7	Alone 11; with others 15; student halls 1; not known 1; not available 7
History of drug use	Yes 16; no 6; not known 3	Yes 2; no 1	6/7 mention drug abuse as a contributing factor	Yes 18; no 8; not known 3; contributing factor 6
Prescribed drug history	Yes 18; no 5; not known 2	Yes 2; no 1	Not available 7	Yes 20; no 6; not known 2; not available 7

Sources: Coroners’ database, NRS.

*No cases in Northern Ireland.

### Circumstances of death

Information in this sub-section is mostly derived from coroners’ data. Only one case in England precisely describes drug-taking location prior to death – at home. Nearly four-fifths (77.8%) died at home versus 11.1% died in hospital; some interventions led to victims being taken to the hospital where they were declared dead on arrival or died subsequently.

About four-fifths (78.9%) were found dead but there were opportunities in several of the remaining cases for interventions: one case was witnessed collapsing; one case was found unresponsive but alive; one case was witnessed to fall out of a window (4.5 m drop); and in one case paramedics were called. However, given that, where known, 41% lived alone, this means that such opportunities were somewhat limited, assuming lone use in the home situation.

### Intentionality/manner of death

For deaths in England and Wales, the Coroner’s Verdict or Conclusion can be used as an indicator of the intentionality of the deceased and/or the manner of their death. Findings for England were accidental 23, suicide 1 and undetermined 1 whilst in Wales there were 3 accidental deaths. For Scotland, ICD-10 codes (World Health Organization (WHO), 1992) have to be relied upon to derive such information. Findings for that country were as follows: 5 accidental (X41), 1 of undetermined intent (Y12), natural causes (J46). Overall, for Great Britain, the key findings are as follows: 31/35 (88.6 %) accidental, 2 (5.7%) undetermined intent/unascertained; 1 (2.9%) natural causes and 1 (2.9%) suicide.

### Causes/mechanisms of death

The underlying (or distal) cause of death is defined here as the last-listed text in Cause 1 of the death certificate. Table 5 provides a breakdown by country and cause. The overwhelming majority (33/35) of cases were ascribed to acute drug toxicity/intoxication: 14 to either diphenidine or MXP alone; 15 to either diphenidine or MXP with other drugs, including 1 case where both molecules were implicated; and 4 cases to other drugs. In addition, cardiac issues, trauma, etc., were also mentioned in the underlying cause of death.

**Table 5.** Underlying cause of death by country.

Country	Underlying cause of death	Number of decedents
England	Acute drug toxicity	23
	MXP/diphenidine alone	9 in one case mirtazapine and fluoxetine cited as cause 2
	MXP/diphenidine and	10
	Other drugs	4
	Cardiac	2 co-cited with MXP/diphenidine toxicity
	Trauma	1
	Unascertained	1
Wales	Acute drug toxicity	3
	MXP/diphenidine alone	2 in one case alcohol cited as cause 2
	MXP/diphenidine and other drugs	1
	Cardiac	1 co-cited with diphenidine toxicity
Scotland	Acute drug toxicity/intoxication	7
	MXP/diphenidine alone	3
	MXP/diphenidine and other drugs	4 in one case with both MXP and diphenidine
	Other drugs	0
Great Britain	Acute drug toxicity	33
	MXP/diphenidine alone	14 in one case, mirtazapine and fluoxetine cited as cause 2, in one case, alcohol cited as cause 2
	MXP/diphenidine and other drugs	15 in one case with both MXP and diphenidine
	Other drugs	4
	Cardiac	2 in one case co-cited with MXP toxicity, in two cases co-cited with diphenidine toxicity
	Trauma	1
	Unascertained	1

### Drug combinations

As far as the authors can establish, at the GB/UK level, the only two molecules of this group of dissociatives involved in death and/or found at post-mortem were diphenidine and MXP. There was only one death (Scotland) where both molecules were implicated in the same death. In Scotland, there were two cases with MXP alone (one had citalopram, cannabis and alcohol also present); one case with diphenidine alone (but temazepam also present). The coroners’ database has records of 9 cases that had MXP (*n* = 8) or diphenidine (*n* = 1) implicated alone. In most cases (23/35; 65.7%), one or other of these two molecules was implicated with other substances (including alcohol) in causing death. The other substances were typically opioids/opiates, benzodiazepines and stimulants (see Tables 6 and 7). The coroner’s database recorded two deaths with diphenidine or MXP detected at post-mortem but no drugs implicated (one death unascertained, one death trauma). In the remaining 26 cases, 9 had MXP/diphenidine implicated alone, and the remaining 17 cases were polydrug ones.

**Table 6.** Drug combinations, Scotland.

Drug	Number of cases where implicated
Eitzolam	3
Morphine	2
Alcohol	1
Ethylphenidate	1
MPA (methiopropramine)	1
Pyrazolam	1

**Table 7.** Drug combinations, England and Wales.

Drug	Number of cases where implicated
Morphine	9
Codeine	7
Alcohol	5
MPA (methiopropramine)	5
Flubromazepam	3
Ketamine	3
Methadone	3
Mirtazapine	3
Amphetamine	2
Dihydrocodeine	2
Ecstasy	2
Etizolam	2
Pregabalin	2
3-FPM	1
4,4-DMAR	1
AH-7921	1
Amitriptyline	1
Citalopram	1
Cocaine	1
Diazepam	1
Ethylphenidate	1
Fluoxetine	1
Lormetazepam	1
Mephedrone	1
Methamphetamine	1
Methoxypiperamide	1
Mexedrone	1
Tramadol	1
Zopiclone	1

### Post-mortem drug levels

Information on *post-mortem* toxicological levels is unavailable to NRS. However, such data are available to the coroners’ database. The available information on blood levels (mg/L) is given in Table 8. There were six cases where MXP alone was implicated for which quantification is available: mean 7.92, median 5.1, range: 1–24. Levels are available for two cases where diphenidine was implicated with other drugs: 0.57, 5.1; and one below the level of quantification (<0.1 mg/L).

**Table 8.** Blood levels reported to the coroners’ database.

MXP implicated alone (*N* = 8)
Six cases with quantifications: 1, 1.6, 2, 8.2, 10.7, 24 mg/L
Median: 5.1 mg/L
Mean: 7.92 mg/L
Range: 1–24 mg/L
MXP implicated with other drugs (*N* = 12)
Two cases with quantifications (and 1 case with a level below the level of quantification)
0.57 mg/L
5.1 mg/L
<0.1 mg/L
Median/mean: 2.84 mg/L
Range: <0.1–5.1 mg/L
MXP detected at post-mortem but not implicated (*N* = 4)
One case with quantification: 0.078 mg/L
Diphenidine
Quantification was performed in two cases. One case 2.4 mg/L (tissue not specified), one case 1.67 mg/L in blood

## Discussion

This is the first study to provide an overview of the global picture of deaths involving diarylethylamines, as well as providing an in-depth description of deaths related to them in the UK. It has used several approaches and a range of sources to establish the overall number of fatalities related to diarylethylamines and to ‘take a deep dive’ into such deaths in the Kingdom, thereby contributing to the consideration of this drug class by the UK’s ACMDs.

### Numbers and trends

Overall, 48 deaths were identified. Most of the cases described here (*n* = 37) occurred in the UK, or more accurately, Great Britain. However, there are cases reported from the United States, mainland Europe and Japan. There may well be cases in other countries that have not been identified or reported.

The majority of deaths occurred in the period 2014–2016, with one outlier in 2019. The case reports and statistics from the STRIDA project in Sweden and the European Drug Emergencies Network Plus (Euro-DEN Plus) project regarding acute intoxications cited by the ACMD (2023b) all occurred during the period 2014–2019; diphenidine and MXP were the only diarylethylamines mentioned. Similar findings for fatalities and acute intoxications are reported by the WHO Expert Committee on Drug Dependence (2021: 78–112) in their critical review reports on diphenidine and MXP.

### Characteristics of decedents

The demographic profile of decedents (majority White males, aged mid-late 30s, unemployed) is broadly similar to the majority of the coroners’ database cases, although the average age of the latter cases is about 44 years (Corkery et al., 2021). These findings are consistent with the information available from the STRIDA (Helander et al., 2015) and Euro-DEN Plus (Wood et al., 2023: 62) projects on 20 acute toxicity cases: most involved diphenidine (*n* = 15); males (*n* = 16); age range 20–39 years.

The overall characteristics of the fatalities examined here match well with those of methoxetamine-related cases previously reported from the NPSAD programme (Chiappini et al., 2015). This is not only in terms of gender (male) and ethnicity (White) of the decedents but also in terms of the characteristics of the deaths (polysubstance, including opioids, benzodiazepines) and manner of death (mostly accidental).

### Circumstances and manner of death

There is little information in the published case reports regarding circumstances leading to death, and this is true of the coroners’ cases analysed above, as is the case with other NPS cases (Yoganathan et al., 2022). Some information is available on acute MXP intoxications: two somnolent males found on the street (Hofer et al., 2014; Lam et al., 2016; a male found in a state of agitation (Chrétien et al., 2018); a male involved in a road traffic collision (Stachel et al., 2016).

Information on the *locus* of death is also largely missing from the published case reports, although one of the Japanese diphenidine cases was found dead on a bed (Kudo et al., 2015). The published MXP fatalities were all UK/GB cases and are reported above.

Information on the intentionality or manner of death is missing from the published case reports. However, in terms of the UK/GB deaths described above, most deaths were accidental in nature, although some were intentional.

### Cause of death and toxicology

Most UK/GB decedents died as a result of acute drug toxicity, as was also likely in the cases of the non-UK fatalities. However, other factors contributed to death, including cardiac issues and trauma; these were also noted in acute intoxications (e.g. Hofer et al., 2014; Stachel et al., 2016; Valli et al., 2017).

Both the WHO (2021) and the ACMD (2023b) reports highlight the fact that most deaths involved polysubstance combinations. In terms of the latter and this study, on which it was partly based, two-thirds of deaths involved other substances, typically opioids/opiates, benzodiazepines and stimulants – echoing what is found in deaths involving other drug classes (Kalk et al., 2022; Rock et al., 2022a, 2022b). Most deaths reported here involved MXP and then diphenidine; there were no cases involving ephenidine, fluorolintane or isophenidine. Polypharmacy is also evident in several of the acute toxicity cases reported in the ACMD report; however, other such cases report only one molecule being detected in the toxicology of patients admitted to the hospital for treatment.

MXP and diphenidine were implicated on their own in about one-third of cases; this suggests that they are relatively toxic. If one were to apply the King and Corkery’s (2018) ‘Index of Fatal Toxicity’ approach, that is, ratio of sole versus all mentions in the cause of death to these findings for both molecules combined, it would yield a ratio of 0.414 (i.e., 12/29, see Table 5). This result is higher than those for mephedrone (0.197), MPA (methiopropamine) (0.207), synthetic cathinones (0.217), piperazines (0.235), cocaine/crack (0.321), benzofurans (0.351) and amphetamine (0.371). The ratio for these two dissociative molecules combined (0.414) is on a par with that for ketamine (0.411) – perhaps suggesting a similar level of toxicity. However, these molecules would not appear to be as toxic as PMA/PMMA (para-methoxyamphetamine/para-methoxy-N-methylamphetamine) (0.497); GHB/GBL – hydroxybutyrate/γ-butyrolactone (0.549); synthetic cannabinoids (0.579); heroin (0.604); and AMT (α-methyltryptamine) (0.630).

The range of diphenidine blood toxicology levels in 14 Swedish non-fatal intoxications was 2–262 ng/ml (Helander et al., 2015); this is a very broad range. However, the present study was able to narrow this range by reporting levels for two fatal cases where diphenidine was implicated with other drugs: 0.57 and 5.1 mg/L. Furthermore, this study presents the first data available on the fatal toxicity of MXP from six cases: mean 7.92, median 5.1, range: 1–24 mg/L (WHO, 2021: 101). These are much lower levels than in the non-fatal cases, supporting the argument that these molecules have high toxicity profiles.

### Implications of study findings

The information above formed a key part of the advice provided by the ACMD to the UK Government. As a result of its deliberations, the ACMD (2023b: 23) recommended.

1. The following compounds (listed under point 2 underneath) should be added to Class B of the Misuse of Drugs Act 1971, consistent with the classification of ketamine and other controlled dissociatives such as methoxetamine and PCP-related materials.
2. As these materials have no medical use, it is recommended that they should be placed in Schedule 1 of the Misuse of Drugs Regulations 2001 (as amended) and added to schedule 1 of the Misuse of Drugs (Designation) (England, Wales and Scotland) Order 2015, Northern Ireland 2001, to which section 7(4) of the Misuse of Drugs Act 1971 applies.
  - Diphenidine
  - Ephenidine
  - Methoxyphenidine (also known as methoxphenidine).

On 10 August 2023, the UK Government accepted both recommendations and indicated that it intended to bring forward legislation to implement them, subject to Parliamentary approval (Home Office, 2023). An Order was laid before Parliament on 21 February 2024 and came into effect on 20 March 2024, making these three molecules Class B drugs (United Kingdom, 2024).

There are several clinical implications arising from the use of these molecules, of which consumers and clinicians should be made aware. Ketamine-like dissociatives, as with those examined here, are one of the classes of NPS commonly associated with the onset of psychopathological consequences (Schifano et al., 2021b). The nasal route of administration means that there is a more rapid absorption and effect, as with esketamine (Chiappini et al., 2025). As lower urinary tract symptoms have been associated with both esketamine and ketamine usage, users and clinicians need to be cognisant that such urological outcomes may result from the use of molecules such as diphenidine and MXP (Chiappini et al., 2025; Schifano et al., 2021a).

### Study strengths and limitations

The research described here made a major contribution to the ACMD’s consideration of this group of molecules. This article puts into the public domain the most detailed and comprehensive information to date bearing on fatalities related to diarylethylamines.

Although it could be argued that the relatively small number of confirmed cases affects statistical reliability, the findings are as robust as can be derived at present. There are some further limitations associated with this research.

Reporting to the coroners’ database is voluntary, and not all cases were reported to it. Some deaths may not have been identified due to the cause of death being described as ‘mixed drug toxicity’ or similar wording being used. There may not have been systematic or routine screening by forensic laboratories for the five molecules examined here; retrospective analyses may reveal more cases. Toxicology levels are not available to NRS, thereby limiting such information from that source. Very limited toxicology reports for deaths attributed solely to diarylethylamines mean that conclusions drawn about toxic levels are not robust.

This issue is redolent of wider issues caused by insufficient details being available about incidents (e.g. *locus* and events leading up to death) and the characteristics of decedents (e.g. ethnicity, socio-economic status), which might provide some insight into the nature of ‘at-risk’ populations.

The fact that most fatalities were polydrug in nature means that it is difficult to isolate complications arising from drug combinations and/or drug-drug interactions, thereby establishing possible causal connections between diarylethylamines use and death.

Limited or insufficient toxicology reports and inconsistencies across cases weaken conclusions about toxic levels for each substance.

### Recommendations

The authors concur with the ACMD’s recommendations and the UK Government’s subsequent legislative action regarding control of the molecules discussed above.

Although these molecules were only apparent during a limited timeframe, it is important that forensic toxicologists, pathologists and clinicians remain aware of their existence and how to respond to incidents appropriately. A quick check of the Internet (open net) at the start of January 2025 indicated that diphenidine, ephenidine and MXP are still being offered for sale as ‘research chemicals’. Diphenidine and MXP were still being discussed on Reddit in late December 2024.

Those responsible for identifying such products should ensure that these molecules are included in their regular drug screens for psychoactive substances and reported to relevant drug surveillance agencies.

## Conclusions

Deaths involving diarylethylamines are relatively rare; 48 were identified by this study, of which 37 occurred in the UK. Methoxphenidine and diphenidine are the only two molecules from this drug class that were implicated in death; they are relatively toxic when compared to most stimulants but on a par with ketamine.

Most deaths identified and reported here took place within a confined timeframe (2014–2016) and geographical setting (mostly Great Britain). Despite these temporal and spatial constraints, as these molecules are still being discussed on drug users’ fora and are still available for purchase online, further deaths may occur. Potentially, other non-fatal adverse effects could still occur, further underlining the need for these molecules to be controlled under the Misuse of Drugs Act 1971.

The socio-demographics of decedents are similar to those found in other stimulant-related fatalities, especially in the UK. The manner and underlying causes of death are also like those of stimulant-related deaths more widely.

It is likely that other fatalities have occurred but have not been identified and/or reported to appropriate agencies. Should further cases become known (e.g. from retrospective analyses of samples), they should be added to this emerging evidence base.

## Supplemental Material

sj-docx-1-jop-10.1177_02698811251349203 – Supplemental material for Deaths related to the use of diarylethylamines, with a focus on the United Kingdom: A systematic review and case series report Supplemental material, sj-docx-1-jop-10.1177_02698811251349203 for Deaths related to the use of diarylethylamines, with a focus on the United Kingdom: A systematic review and case series report by John Martin Corkery, Caroline Copeland and Fabrizio Schifano in Journal of Psychopharmacology

## References

[bibr1-02698811251349203] ACMD (2023a) Letter from ACMD to the Home Secretary: ACMD Review of Diphenidine. London, UK: Home Office. Available at: https://assets.publishing.service.gov.uk/media/646f21e47dd6e7000ca9b36a/Diphenidine_Covering_Letter_FINAL_DRAFT.pdf (accessed 7 July 2024).

[bibr2-02698811251349203] ACMD (2023b) ACMD Report – A Review of the Evidence on the Use and Harms of Diphenidine and Other Related Substances. London, UK: Advisory Council on the Misuse of Drugs. Available at: https://assets.publishing.service.gov.uk/media/646f41f6243157000c6f4275/ACMD_Diphenidine_Report_COPY_FOR_ACCESSIBILITY_CHECKING_-_FINAL.pdf (accessed 7 July 2024).

[bibr3-02698811251349203] Chiappini S Claridge H Corkery JM , et al (2015) Methoxetamine-related deaths in the UK: An overview. Hum Psychopharmacol 30: 244–248. 26216557 10.1002/hup.2422

[bibr4-02698811251349203] Chiappini S Guirguis A Schifano N , et al (2025) Comparative safety of prescribed Esketamine and ketamine in relation to renal and urinary disorders: A pharmacovigilance perspective. Prog Neuropsychopharmacol Biolog Psychiatry 136: 111213. 10.1016/j.pnpbp.2024.111213 39647692

[bibr5-02698811251349203] Chrétien B Bourgine J Hamel Sénécal L , et al (2018) Severe serotonin syndrome in an autistic new psychoactive substance user after consumption of pills containing methoxphenidine and α-methyltryptamine. J Clin Psychopharmacol 38: 94–96. 29210865 10.1097/JCP.0000000000000816

[bibr6-02698811251349203] Corkery JM Hung WC Claridge H , et al (2021) Recreational ketamine-related deaths notified to the National Programme on Substance Abuse Deaths, England, 1997–2019. J Psychopharmacol 35: 1324–1348. 34092131 10.1177/02698811211021588 PMC8600594

[bibr7-02698811251349203] Courts and Tribunals Judiciary (2015) Prevention of future death reports, Catherine Findlay, Ref: 2015-0372. Available at: https://www.judiciary.uk/prevention-of-future-death-reports/catherine-findlay/ (accessed 29 July 2024).

[bibr8-02698811251349203] Deen AA Claridge H Treble RD , et al (2021) Deaths from novel psychoactive substances in England, Wales and Northern Ireland: Evaluating the impact of the UK psychoactive substances act 2016. J Psychopharmacol 35: 1315–1323. 34182812 10.1177/02698811211026645 PMC8600590

[bibr9-02698811251349203] Eiden C Leone-Burgos S Serre A , et al (2018) Ephenidine, diphenidine, and methoxphenidine complications reported to the French Addictovigilance Network. Fundam Clin Pharmacol 32: 654–662. 29956843 10.1111/fcp.12395

[bibr10-02698811251349203] Elliott SP Brandt SD Wallach J , et al (2015) First reported fatalities associated with the ‘research chemical’ 2-methoxydiphenidine. J Anal Toxicol 39: 287–293. 25698777 10.1093/jat/bkv006

[bibr11-02698811251349203] Elliott S Sedefov R Evans-Brown M (2018) Assessing the toxicological significance of new psychoactive substances in fatalities. Drug Test Anal 10: 120–126. 28635164 10.1002/dta.2225

[bibr12-02698811251349203] Goddard B (2015) Father sends ‘legal highs’ danger alert after son’s death. Powys County Times, 18 June. Available at: https://www.countytimes.co.uk/news/15834896.father-sends-legal-highs-danger-alert-after-sons-death/ (accessed 29 July 2024).

[bibr13-02698811251349203] Grumann C Vogt S Huppertz LM , et al (2017) A case of methoxphenidine intoxication. GTFCh-Symposium Poster. P.27. Toxichem Krimtech 84: 102.

[bibr14-02698811251349203] Hasegawa K Wurita A Minakata K , et al (2015) Postmortem distribution of AB-CHMINACA, 5-fluoro-AMB, and diphenidine in body fluids and solid tissues in a fatal poisoning case: Usefulness of adipose tissue for detection of the drugs in unchanged forms. Forensic Toxicol 33: 45–53.

[bibr15-02698811251349203] Helander A Beck O Bäckberg M (2015) Intoxications by the dissociative new psychoactive substances diphenidine and methoxphenidine. Clin Toxicol (Phila) 53: 446–453. 25881797 10.3109/15563650.2015.1033630

[bibr16-02698811251349203] Hofer KE Degrandi C Müller DM , et al (2014) Acute toxicity associated with the recreational use of the novel dissociative psychoactive substance methoxphenidine. Clin Toxicol (Phila) 52: 1288–1291. 25350467 10.3109/15563650.2014.974264

[bibr17-02698811251349203] Home Office (2023) The government response to the ACMD advice on diphenidine and related substances. London, UK: Home Office. Available at: https://assets.publishing.service.gov.uk/government/uploads/system/uploads/attachment_data/file/1176976/Letter_to_ACMD_-_diphenidine.pdf (accessed 3 August 2024).

[bibr18-02698811251349203] Kalk NJ Chiu CT Sadoughi R , et al (2022) Fatalities associated with gabapentinoids in England (2004-2020). Br J Clin Pharmacol 88: 3911–3917. 35435281 10.1111/bcp.15352 PMC9543893

[bibr19-02698811251349203] Kang H Park P Bortolotto ZA , et al (2017) Ephenidine: A new psychoactive agent with ketamine-like NMDA receptor antagonist properties. Neuropharmacology 112: 144–149. 27520396 10.1016/j.neuropharm.2016.08.004 PMC5084681

[bibr20-02698811251349203] King LA Corkery JM (2018) An index of fatal toxicity for new psychoactive substances. J Psychopharmacol 32: 793–801. 29482434 10.1177/0269881118754709

[bibr21-02698811251349203] Kinoshita H Tanaka N Takakura A , et al (2017) An autopsy case of death by combined use of benzodiazepines and diphenidine. Soudni Lekarstvi 62: 40–43. 29227117

[bibr22-02698811251349203] Kudo K Usumoto Y Kikura-Hanajiri R , et al (2015) A fatal case of poisoning related to new cathinone designer drugs, 4-methoxy PV8, PV9, and 4-methoxy PV9, and a dissociative agent, diphenidine. Legal Med (Tokyo) 17: 421–426. 10.1016/j.legalmed.2015.06.005 26162997

[bibr23-02698811251349203] Kusano M Zaitsu K Taki K , et al (2018). Fatal intoxication by 5F-ADB and diphenidine: Detection, quantification, and investigation of their main metabolic pathways in humans by LC/MS/MS and LC/Q-TOFMS. Drug Test Anal 10: 284–293. 28544560 10.1002/dta.2215

[bibr24-02698811251349203] Lam RP Yip WL Tsui MS , et al (2016) Severe rhabdomyolysis and acute kidney injury associated with methoxphenidine. Clin Toxicol (Phila) 54: 464–465. 27007750 10.3109/15563650.2016.1157724

[bibr25-02698811251349203] Logan BK Mohr ALA Friscia M , et al (2017) Reports of adverse events associated with use of novel psychoactive substances, 2013–2016: A review. J Anal Toxicol 41: 573–610. 28459969 10.1093/jat/bkx031

[bibr26-02698811251349203] Minakata K Yamagishi I Nozawa H , et al (2016) Semiquantitation of diphenidine in tissue sections obtained from a human cadaver in a poisoning case by direct MALDI-QTOF mass spectrometry. Forensic Toxicol 34: 151–157.

[bibr27-02698811251349203] Morris H Wallach J (2014) From PCP to MXE: A comprehensive review of the non-medical use of dissociative drugs. Drug Test Anal 6: 614–632. 24678061 10.1002/dta.1620

[bibr28-02698811251349203] Rock KL Reynolds LM Rees P , et al (2022a) Highlighting the hidden dangers of a ‘weak’ opioid: Deaths following use of dihydrocodeine in England (2001-2020). Drug Alcohol Depend 233: 109376. 35248998 10.1016/j.drugalcdep.2022.109376

[bibr29-02698811251349203] Rock KL Englund A Morley S , et al (2022b) Can cannabis kill? Characteristics of deaths following cannabis use in England (1998-2020). J Psychopharmacol 36: 1362–1370. 35946604 10.1177/02698811221115760 PMC9716494

[bibr30-02698811251349203] Sahai MA Davidson C Dutta N , et al (2018) Mechanistic insights into the stimulant properties of novel psychoactive substances (NPS) and their discrimination by the dopamine transporter – In silico and in vitro exploration of dissociative diarylethylamines. Brain Sci 8: 63. 29642450 10.3390/brainsci8040063 PMC5924399

[bibr31-02698811251349203] Saint-Martin P Gambier A Deveaux M , et al (2017) Death due to mixed drug intoxication including multiple new psychoactive substances: A case report. Abstract, p. 13, for IAFS 2017. Inter-Professional Collaboration in Forensic Science. Toronto, Canada, 21-25 August 2017. Forensic Sci Int 277: 1–257.

[bibr32-02698811251349203] Schifano F Napoletano F Chiappini S , et al (2019) New psychoactive substances (NPS), psychedelic experiences, and dissociation: Clinical and clinical pharmacological issues. Curr Addict Rep 6: 140–152.

[bibr33-02698811251349203] Schifano N Chiappini S Castiglione F , et al (2021a) Is medicinal ketamine associated with urinary dysfunction issues? Assessment of both the European Medicines Agency (EMA) and the UK Yellow Card Scheme pharmacovigilance database-related reports. Lower Urinary Tract Sympt 13: 230–237. 10.1111/luts.12355 33037767

[bibr34-02698811251349203] Schifano F Napoletano F Chiappini S , et al (2021b) New/emerging psychoactive substances and associated psychopathological consequences. Psychol Med 51: 30–42. 31327332 10.1017/S0033291719001727

[bibr35-02698811251349203] Stachel N Jacobsen-Bauer A Skopp G (2016) A methoxydiphenidine-impaired driver. Int J Legal Med 130: 405–409. 26482953 10.1007/s00414-015-1280-5

[bibr36-02698811251349203] Sussex World (2014) Coroner warns of dangers of ‘legal high’ drug. Sussex World, 27 November. Available at: https://www.sussexexpress.co.uk/news/coroner-warns-of-dangers-of-legal-high-drug-2255677 (accessed 29 July 2024).

[bibr37-02698811251349203] United Kingdom (2024) The Misuse of Drugs Act 1971 (Amendment) Order 2024 (SI 2024/190). Available at: https://www.legislation.gov.uk/uksi/2024/190/introduction/made (accessed 3 August 2024).

[bibr38-02698811251349203] Valli A Lonati D Locatelli CA , et al (2017) Analytically diagnosed intoxication by 2-methoxphenidine and flubromazepam mimicking an ischemic cerebral disease. Clin Toxicol (Phila) 55: 611–612. 28452234 10.1080/15563650.2017.1286016

[bibr39-02698811251349203] Van Hout MC Hearne E (2015) “Word of mouse”: Indigenous harm reduction and online consumerism of the synthetic compound methoxphenidine. J Psychoact Drugs 47: 30–41. 10.1080/02791072.2014.974002 25715070

[bibr40-02698811251349203] Wallach J Colestock T Agramunt J , et al (2019) Pharmacological characterizations of the ‘legal high’ fluorolintane and isomers. Eur J Pharmacol 857: 172427. 31152702 10.1016/j.ejphar.2019.172427 PMC6899220

[bibr41-02698811251349203] Wallach J Kang H Colestock T , et al (2016) Pharmacological investigations of the dissociative ‘legal highs’ diphenidine, methoxphenidine and analogues. PLoS One 11: e0157021. 10.1371/journal.pone.0157021 PMC4912077 27314670

[bibr42-02698811251349203] Wallach J Kavanagh PV McLaughlin G , et al (2015) Preparation and characterization of the ‘research chemical’ diphenidine, its pyrrolidine analogue, and their 2,2-diphenylethyl isomers. Drug Test Anal 7: 358–367. 25044512 10.1002/dta.1689

[bibr43-02698811251349203] WHO (1992) The ICD-10 Classification of Mental and Behavioural Disorders: Clinical Descriptions and Diagnostic Guidelines. Geneva, Switzerland: World Health Organization. Available at: https://apps.who.int/iris/handle/10665/37958 (accessed 25 February 2024).

[bibr44-02698811251349203] WHO Expert Committee on Drug Dependence (2021) WHO Expert Committee on Drug Dependence: Forty-Third Report (WHO Technical Report Series, No. 1034). Geneva, Switzerland: World Health Organization. Available at: https://iris.who.int/bitstream/handle/10665/340712/9789240023024-eng.pdf?sequence=1 (accessed 1 August 2024).

[bibr45-02698811251349203] Winter A (2016) Man died after taking former legal high MXP, inquest hears. Bournemouth Daily Echo, 26 August. Available at: https://www.bournemouthecho.co.uk/news/14705056.man-died-after-taking-former-legal-high-mxp-inquest-hears/ (accessed 29 July 2024).

[bibr46-02698811251349203] Wood DM Dines AM Liechti ME , et al (2023) Diphenidine, methoxyphenidine and related 1,2-diarylethylamine acute toxicity reported to the Euro-DEN Plus project. Abstract 128, 43rd International Congress of the European Association of Poisons Centres and Clinical Toxicologists (EAPCCT), 23-26 May 2023, Palma de Mallorca, Spain. Clin Toxicol (Phila) 61: 62.

[bibr47-02698811251349203] Yoganathan P Claridge H Chester L , et al (2022) Synthetic cannabinoid-related deaths in England, 2012-2019. Cannabis Cannabinoid Res 7: 516–525. 33998886 10.1089/can.2020.0161 PMC9418359

