# Supplementary material for: Deaths related to the use of diarylethylamines, with a focus on the United Kingdom: A systematic review and case series report
Source: J Psychopharmacol. 2025 Jul 1;40(2):325–38. doi: 10.1177/02698811251349203 (PMC13222385; doi:10.1177/02698811251349203)
Supplement: sj-docx-1-jop-10.1177_02698811251349203 – Supplemental material for Deaths related to the use of diarylethylamines, with a focus on the United Kingdom: A systematic review and case series report [file sj-docx-1-jop-10.1177_02698811251349203.docx]

**Deaths related to the use of diarylethylamines, with a focus on the United Kingdom: a systematic review and case-series report – Supplementary material**

*Systematic review of academic literature*

The first author conducted searches on 29 and 30 August 2022 of bibliographic databases (PubMed, Scopus, Google Scholar) using the following sets of terms, including the variant chemical names of the five molecules:

( ((overdose) or (death) or (fatal*) or (toxic*) or (poison*)) and ((2-methoxy-diphenidine) or (2-MXP) or ((±)-1-[1-(2-methoxyphenyl)-2-phenylethyl] piperidine) or (2-Meo-diphenidine) or (Diphenidine) or (DPH) or (Ephenidine) or (Methoxphenidine) or (MXP) or (3-methoxy-diphenidine) or (3-MXP) or (4-methoxy-diphenidine) or (4-MXP) or (2-Cl-diphenidine) or (2-Cl-DPH)) )

((overdose) or (death) or (fatal*) or (toxic*) or (poison*)) and (Diphenidine)

((overdose) or (death) or (fatal*) or (toxic*) or (poison*)) and (Ephenidine)

((overdose) or (death) or (fatal*) or (toxic*) or (poison*)) and (Methoxphenidine)

((overdose) or (death) or (fatal*) or (toxic*) or (poison*)) and (MXP)

All ‘hits’ from these searches were firstly examined for relevance by considering their titles and abstracts (where available). Those considered to be possibly relevant were then read and their contents scanned for information relevant to the study. Finally, the reference lists were examined to identify any other possible references or sources of information.

All types of research reports were included, published in any language, from any country, and any period. The only exclusion criterion was if the subjects were not human.

Other sources surveyed, based on the authors’ knowledge were: European Monitoring Centre for Drugs and Drug Addiction (EMCDDA) risk assessments <https://www.euda.europa.eu/activities/ews/risk-assessment-new-psychoactive-substances-nps_en#section4>; EUropean-wide, Monitoring, Analysis and knowledge Dissemination on Novel/Emerging pSychoactiveS (EU-MADNESS) cases <https://x.com/eu_madness?lang=en-GB>, <https://www.facebook.com/EUmadnessproject/?locale=hu_HU>; Scottish Fatal Accident Inquiries <https://www.scotcourts.gov.uk/search-judgments/fatal-accident-inquiries>; and the Chief Coroners website - Regulation 28 Reports to Prevent Future Deaths <https://www.judiciary.uk/courts-and-tribunals/coroners-courts/reports-to-prevent-future-deaths/>.

At the time of writing (early May 2025), having repeated the above searches, the authors are unaware of any later publications or reports relating to additional cases.
